# Oestradiol dynamics after polytrauma are linked to injury severity and adverse outcomes

**DOI:** 10.3389/fendo.2026.1878946

**Published:** 2026-08-03

**Authors:** Gregor Bielesch, Conrad Ketzer, Viktoria Hess, Luca Kirstein, Frederik Aasen-Hartz, Tobias Resch, Peter Biberthaler, Marc Hanschen, Olivia Bohe

**Affiliations:** Department of Trauma Surgery, TUM Klinikum rechts der Isar, Technical University of Munich, Munich, Germany

**Keywords:** Injury Severity Score, ISS, oestradiol, organ failure, outcome prediction, polytrauma, reproductive hormones

## Abstract

**Introduction:**

Women show lower mortality and fewer complications than men after severe polytrauma, but underlying mechanisms remain unclear. In this context, reproductive hormones have gained increasing attention. In particular, oestradiol is controversially discussed: experimental studies suggest protective effects, whereas clinical studies link elevated levels to systemic inflammation and adverse outcomes.

**Methods:**

Therefore, in this prospective cohort study, patients with polytrauma(injury severity score ≥16) were enrolled. Hormones (oestradiol, progesterone, testosterone, luteinising hormone [LH], follicle-stimulating hormone [FSH]) were measured on admission and daily on days 1–5. Longitudinal changes were analysed with repeated-measures models. Associations with injury severity and clinical outcomes (organ failure, length of stay, mortality) were assessed using correlation and regression analyses adjusted for relevant confounders.

**Results:**

128 patients, progesterone, testosterone, LH, and FSH declined significantly over time, whereas oestradiol showed an early rise with a peak around the second day followed by a decline. Sex-specific differences were observed for testosterone, LH, and FSH, but not for oestradiol. Elevated oestradiol levels and post-traumatic increases were independently associated with injury severity and adverse outcomes, including organ failure, longer stay and mortality, after adjustment for injury severity. These findings were consistent across different oestradiol metrics (peak, area under the curve, and absolute change).

**Discussion:**

be an integral component of the early systemic response to polytrauma and are associated with injury severity and adverse outcomes. These findings challenge the concept of oestradiol as a uniformly protective factor and support its role as a marker of post-traumatic stress and systemic dysregulation.

## Graphical Abstract

**Figure:**
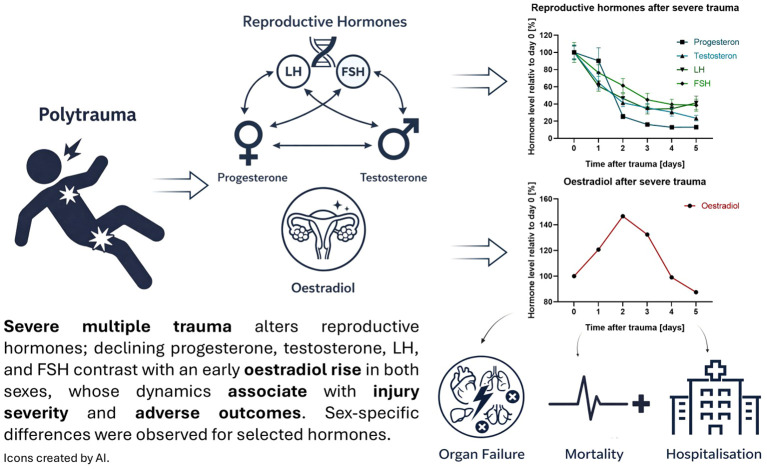

## 1 Introduction

Polytrauma represents a major global health burden and is a leading cause of death and disability worldwide, particularly among younger individuals. Mortality in polytrauma is highest in the early post-injury period, with overall rates ranging from 15% to 27% in recent large cohorts (1, 2). Polytrauma is defined as a life-threatening injury affecting at least two body regions or organ systems (3). An Injury Severity Score (ISS) of ≥16 is commonly used to define severe cases and identify patients at high risk of adverse outcomes (1, 4).

Deaths following polytrauma can be broadly classified into early and late phases according to the time of occurrence (5). Early mortality is predominantly driven by the severity of the initial injury and uncontrollable haemorrhage, whereas late mortality is more commonly related to secondary complications, including infections, sepsis, and multiple organ dysfunction or failure (MOF) (1, 2, 6–8). The development of organ dysfunction and late mortality after polytrauma has been extensively investigated (9–11). Various mechanisms have been proposed to explain this phenomenon, including immunological imbalance (12), dysregulated inflammatory responses, and trauma-associated thromboembolic alterations (13, 14). All of these conditions are thought to contribute to post-traumatic morbidity and mortality.

In this context, sex-specific differences in outcome after polytrauma have repeatedly been reported (15, 16). Overall, male patients appear to be more susceptible to complications such as MOF or sepsis, and show higher short-term mortality following severe injury (17, 18). In contrast, female patients, particularly older women, have been described to exhibit improved survival and lower rates of certain complications in several cohorts, although findings are not entirely consistent across studies (19, 20). These observed differences have fuelled ongoing interest in biological sex as a modifier of trauma outcome.

One potential explanation for sex-related differences lies in the markedly different endocrine environments between men and women. Premenopausal women exhibit substantially higher circulating concentrations of oestradiol and progesterone (21–24), while levels of luteinising hormone (LH) and follicle-stimulating hormone (FSH) fluctuate across the menstrual cycle and rise after menopause (21, 25–27). Men, however, have higher circulating testosterone concentrations (21, 28–30).

Following polytrauma, the endocrine milieu changes profoundly. Suppression of the hypothalamic–pituitary–gonadal axis is well described, resulting in a general decline in reproductive hormone concentrations. Testosterone concentrations decline rapidly and remain suppressed for weeks to months after injury, a pattern associated with increased catabolism, immune dysfunction, and impaired recovery (31–34). Conversely, some studies suggest that initially elevated testosterone concentrations may be linked to exaggerated inflammatory responses and an increased risk of MOF and infectious complications (35, 36). Progesterone levels also decline after trauma, reflecting central suppression and altered peripheral metabolism (32, 37, 38). Although progesterone exhibits anti-inflammatory, anti-apoptotic, and blood–brain barrier–stabilising effects in experimental settings, clinical trials investigating exogenous progesterone administration after traumatic brain injury (TBI) have failed to demonstrate a clear improvement in outcome (39, 40). Gonadotropins (LH and FSH) similarly decline after severe injury, which may contribute to amenorrhoea and anovulation in female patients and may adversely affect long-term recovery (35, 41–44).

Among reproductive hormones, oestradiol has attracted particular interest in the trauma setting. Traditionally, oestradiol has been regarded as protective, largely based on experimental animal studies demonstrating beneficial effects after trauma-haemorrhage (45). These include anti-inflammatory, anti-apoptotic, and organ-protective effects (46). Collectively, these mechanisms have been associated with improved organ function and survival in experimental models.

Recent clinical data have provided a more complex and partly contrasting picture. In the setting of severe trauma, oestradiol concentrations may increase despite suppression of gonadotropins, primarily due to enhanced peripheral aromatisation of androgens (47). To date, this concept is supported by only a limited number of clinical investigations, comprising six studies across trauma-related populations. In patients with polytrauma, two studies have reported associations between oestradiol concentrations and injury severity (33, 42). One study reported increases in oestradiol levels over early post-injury intervals that were linked to a higher risk of MOF or mortality (34). The available evidence is largely derived from single-centre cohorts and is subject to several methodological limitations. All analyses relied on single measurements or two early sampling time points, precluding a detailed assessment of individual hormone trajectories. Moreover, outcome analyses were restricted to a single endpoint, MOF, without consideration of a broader range of clinically relevant adverse outcomes.

Evidence from patients with severe TBI further suggests that elevated oestradiol levels, increasing oestradiol trajectories, or enhanced peripheral aromatisation may be associated with increased mortality and impaired long-term functional recovery (35, 47, 48). Nevertheless, these findings originate from isolated TBI cohorts rather than polytrauma populations and employed grouped or averaged hormone measures over several days. As a result, temporal variability at the individual level and interactions with overall injury severity were not consistently accounted for. Beyond trauma-specific populations, elevated oestradiol concentrations have also been observed in critically ill intensive care patients, where higher levels were reported in non-survivors (49). Taken together, these findings suggest a complex role of oestradiol in the response to severe trauma that is not yet fully understood.

Against this background, the primary aim of the present study was to investigate the association between oestradiol concentrations, injury severity as quantified by the ISS, and adverse clinical outcomes following polytrauma. Secondary objectives were to characterise temporal patterns of oestradiol after injury and to explore potential sex-specific differences in hormonal responses and outcome associations.

## 2 Materials and methods

### 2.1 Data collection

This prospective, non-interventional study enrolled severely injured patients with an ISS ≥ 16 who presented to the trauma room of a Level 1 trauma centre in Germany between April 2023 and December 2025. If unclear upon admission, ISS was calculated retrospectively and subsequently verified. Exclusion criteria comprised age under 18 years, pregnancy, inter-hospital transfers, and death within the first 48 hours of admission. Written informed consent was obtained from all patients or their relatives during the clinical course. The study was reviewed and approved by the local ethics committee of the Technical University of Munich (Trial No: 2022-687-S-SR) on 13 March 2023.

Clinical data collected for each patient included age, sex, pre-existing medical conditions, mechanism of injury, and detailed injury patterns, with severity classified according to the Abbreviated Injury Scale (AIS). Laboratory results and treatment data were recorded, such as damage-control surgical procedures and blood product transfusions. Outcome measures comprised organ failure (OF), raised intracranial pressure, infectious complications, in-hospital mortality, and length of stay (LOS) in the intensive care unit (ICU) and in hospital. OF was defined as an increase in the Sequential Organ Failure Assessment (SOFA) score of ≥2 points during the initial hospitalisation, considering respiratory, cardiovascular, central nervous, hepatic and renal function.

All patients who regained full consciousness during treatment were asked to voluntarily complete a questionnaire on their hormonal status (Supplement 1: Questionnaire). If patients were unable to provide this information themselves, relatives or legal representatives were asked to complete the questionnaire where appropriate.

### 2.2 Hormone testing

Hormone measurements were performed on routinely obtained blood samples collected in the trauma room and on days 1–5 after injury. In addition to parameters determined as part of standard clinical care, serum concentrations of 17β oestradiol (subsequently referred to as oestradiol), progesterone, testosterone, LH and FSH were measured. If a patient received fewer than 3 routine blood samples during the study period or was discharged/transferred before the 5th day after trauma, the patient was subsequently excluded from the study.

Blood was drawn and collected into commercially available Serum S-Monovettes^®^ (Sarstedt AG & Co., Nümbrecht, Germany). Serum concentrations of 17β-oestradiol, progesterone, testosterone, LH, and FSH were determined using electrochemiluminescence immunoassays (ECLIA) on a Cobas 8000 analyser (e 801 module; Roche Diagnostics, Mannheim, Germany). The following reagent kits were used according to the manufacturer’s instructions: cobas e pack E2 3 (oestradiol), PROG 3 (progesterone), TESTO 2 (testosterone), LH, and FSH. Validation of the assays was based on IVDR-approved commercial kits provided by the manufacturer. In accordance with internal laboratory standards, samples were measured within 48 hours of blood sampling.

### 2.3 Statistical analysis

Statistical analyses were performed using GraphPad Prism 8 (version 8.0.2.263; GraphPad Software Inc., Boston, MA, USA) and RStudio IDE (version 2026.01.0 + 392; Posit Software, PBC, Boston, MA, USA). Sample size estimation was based on a simulation-based power analysis assuming a logistic regression model with adverse events as a binary outcome and an expected incidence of 40%. Oestradiol was modelled as a continuous, standardised variable, with effect estimates expressed as odds ratios per one standard deviation increase and adjusted for biological sex. Under the assumption of a clinically relevant effect size of OR = 1.8 per one standard deviation increase in oestradiol, a total sample size of n = 100 yielded an estimated power of approximately 79% (95% CI 78–80%) at a two-sided significance level of α = 0.05, based on 5,000 Monte Carlo simulations.

Descriptive statistics were used to characterise the study cohort and hormone concentrations, with continuous variables reported as mean ± standard deviation. Hormone concentrations were logarithmically transformed prior to analysis to account for skewed distributions, and analyses were stratified by biological sex where applicable. Hormone levels were additionally summarised using peak values, area under the curve (AUC) calculated by the trapezoidal rule, and absolute change from baseline at day 0 (Δ). Oestradiol peak values were not assigned if no measurements were available on days 2 and 3. Longitudinal hormone dynamics were analysed using mixed-effects models and repeated-measures analysis of variance, with *post-hoc* testing performed to identify significant changes over time.

Associations between hormone measures and clinical parameters were explored using Pearson or Spearman correlation coefficients and visualised using correlation heatmaps. Given the exploratory nature of these analyses, no formal adjustment of the significance level was applied; instead, associations identified in the heatmaps were subsequently evaluated using confirmatory regression and receiver operating characteristic (ROC) analyses.

Associations with outcome parameters were assessed using simple and multiple linear regression models, with regression coefficients, 95% CIs, p-values, and the Akaike information criterion (AIC) reported as appropriate. Dichotomous outcomes were analysed using univariable and multivariable binary logistic regression models, and model discrimination was assessed using ROC curve analyses.

Sensitivity analyses included comparisons of different hormonal summary measures, exclusion of patients receiving exogenous hormone therapy, and evaluation of missing data handling using appropriate imputation procedures. All statistical tests were two-sided, and p-values < 0.05 were considered statistically significant.

## 3 Results

### 3.1 Patient characteristics

A total of 128 patients with severe trauma (ISS ≥ 16) were included. Baseline characteristics are presented in **Table 1**. The cohort was predominantly male. Overall injury severity was high (ISS 26.5 ± 10.3) and comparable between sexes (27.4 ± 10.3 in women vs. 26.1 ± 10.4 in men; p = 0.373). Organ failure occurred in 39.1% of patients, and in-hospital mortality was 16.4%. No significant sex-specific differences were observed in clinical outcomes. Hormonal status questionnaires were completed for 89 patients (69.5%). Of these, 67 questionnaires were completed by the patients themselves and 22 by relatives or legal representatives.

**Table 1:** Baseline characteristics, injury patterns, treatment, and clinical outcomes of the study population.

Characteristic	*n* (%)	Mean ± SD
Patient characteristics		
Study population (N)	128	
Age [years]		54.9 ± 20.0
Sex		
Male patients *n*, age [years]	88 (68.9%)	62.2 ± 18.8
Female patients *n*, age [years]	40 (31.3%)	51.6 ± 20.2
Hormonal status in females		
Premenopausal	9 (22.5%)	
Postmenopausal	29 (72.5%)	
Unknown	2 (5.0%)	
Current hormone therapy		
Oestrogen-containing therapy	2 (1.6%)	
Anti-oestrogenic therapy	1 (0.8%)	
ASA score ≥ 3	23 (18.0%)	
Injury		
Mechanism of injury		
Traffic accidents	65 (50.7%)	
Falls	58 (45.3%)	
Penetrating injuries (gunshot/stab wounds)	3 (2.3%)	
Blunt assaults	2 (1.6%)	
Injury pattern (AIS ≥ 1)		
Head/neck injury (AIS)	100 (78.1%)	3.7 ± 1.2
Face injury (AIS)	60 (46.8%)	2.1 ± 0.8
Chest injury (AIS)	65 (50.1%)	3.2 ± 1.2
Abdomen or pelvic organ injury (AIS)	54 (42.2%)	3.2 ± 1.0
Extremity injury (AIS)	59 (46.1%)	2.4 ± 0.7
ISS		26.5 ± 10.3
Treatment and outcome		
Length of stay (LOS)		
ICU length of stay [days]	96 (75%)	11.6 ± 13.2
Hospital length of stay [days]		19.2 ± 14.9
Treatment		
Need for damage control surgery	91 (71.1%)	
Need for blood product transfusion(s)	58 (45.3%)	
Organ failure (OF)	50 (39.1%)	
Multiple organ failure (MOF)	7 (5.5%)	
In-hospital mortality	21 (16.4%)	
Time to death [days]		13.3 ± 16.0

Data are presented as n (%) or mean ± SD.

AIS, Abbreviated Injury Scale; ISS, Injury Severity Score; ASA score, American Society of Anaesthesiologists physical status classification; ICU, intensive care unit; LOS, length of stay; OF, organ failure; MOF, multiple organ failure.

### 3.2 Temporal changes in reproductive hormone levels stratified by sex

**Figure 1:**
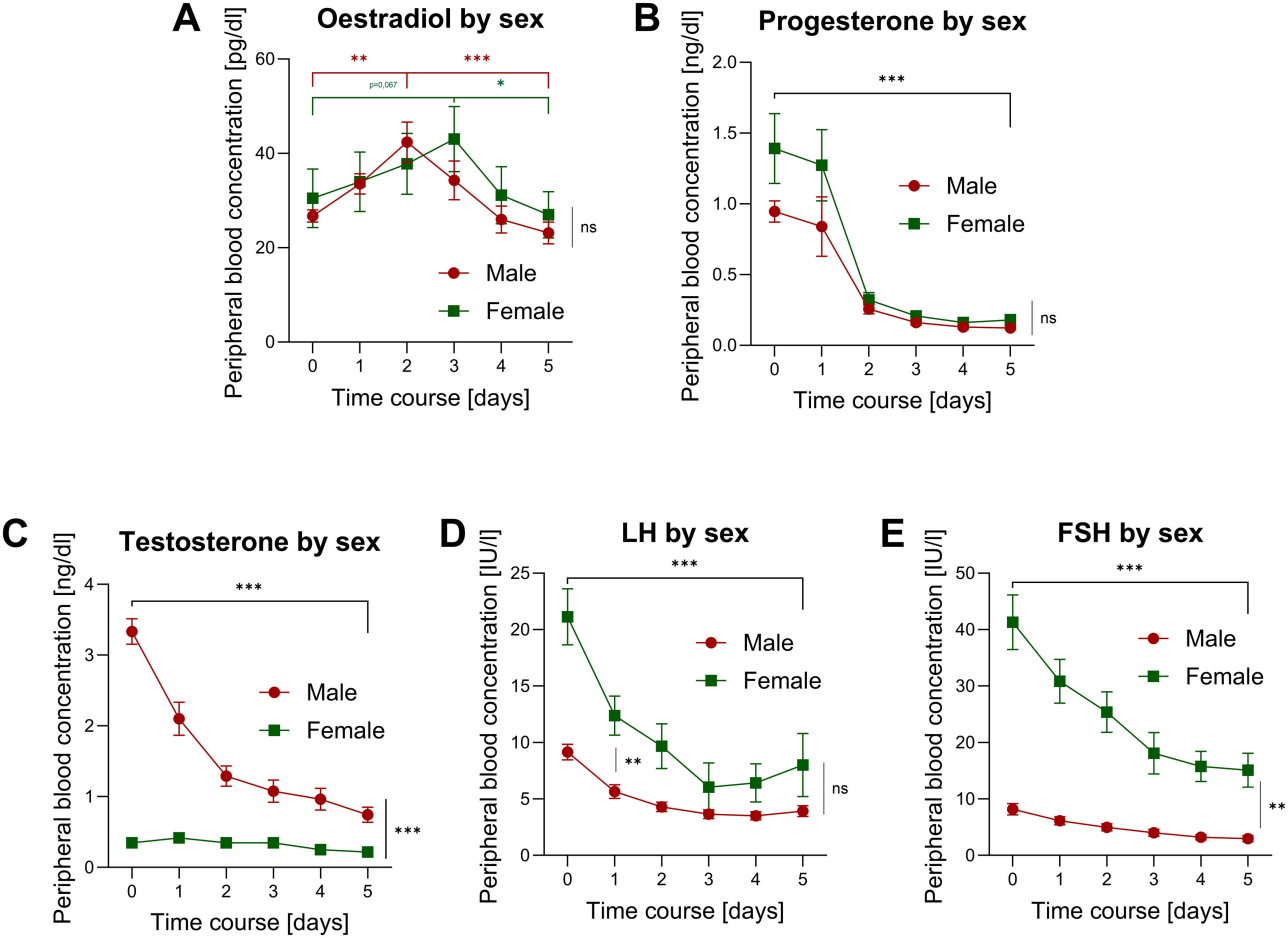
Temporal dynamics of reproductive hormones during the first five days after polytrauma, stratified by sex. Peripheral blood concentrations of 17β-oestradiol **(A)**, progesterone **(B)**, testosterone **(C)**, luteinising hormone [LH, **(D)**], and follicle-stimulating hormone [FSH, **(E)**] were measured on admission (day 0) and daily on days 1–5. Female patients receiving exogenous hormone therapy were excluded (final cohort n = 125). Data are shown as mean ± SD (men, red; women, green). Longitudinal changes were analysed using repeated-measures models. Asterisks above the curves indicate statistically significant within-group changes over time for male patients (red), female patients (green) or both sexes (black), whereas asterisks displayed at the right margin of each panel denote significant differences between sexes (*p < 0.05, **p < 0.01, ***p < 0.001); “ns” denotes non-significant differences.

Longitudinal analyses of reproductive hormone concentrations during the first five days after polytrauma were performed in 125 patients after exclusion of women receiving exogenous hormone therapy (**Figure 1**). Temporal changes were assessed using mixed-effects and repeated-measures models.

Oestradiol showed a distinct temporal pattern (**Figure 1A**), with an early increase after trauma (overall and men p < 0.001; women p = 0.067), peaking at day 2 in men and day 3 in women, followed by a significant decline towards day 5 in both sexes (p < 0.05). No consistent sex differences in oestradiol concentrations were observed. Premenopausal women had higher oestradiol levels on day 1 compared with postmenopausal women (p = 0.033), with no differences thereafter (**Supplementary Figure 1**).

In contrast, progesterone, testosterone, LH, and FSH concentrations declined significantly over time in both sexes (**Figures 1B–E**). Sex-specific differences were observed for testosterone, FSH, and early LH levels, whereas overall temporal trends were comparable between sexes.

### 3.3 Correlation analysis of oestradiol parameters and clinical variables

**Figure 2:**
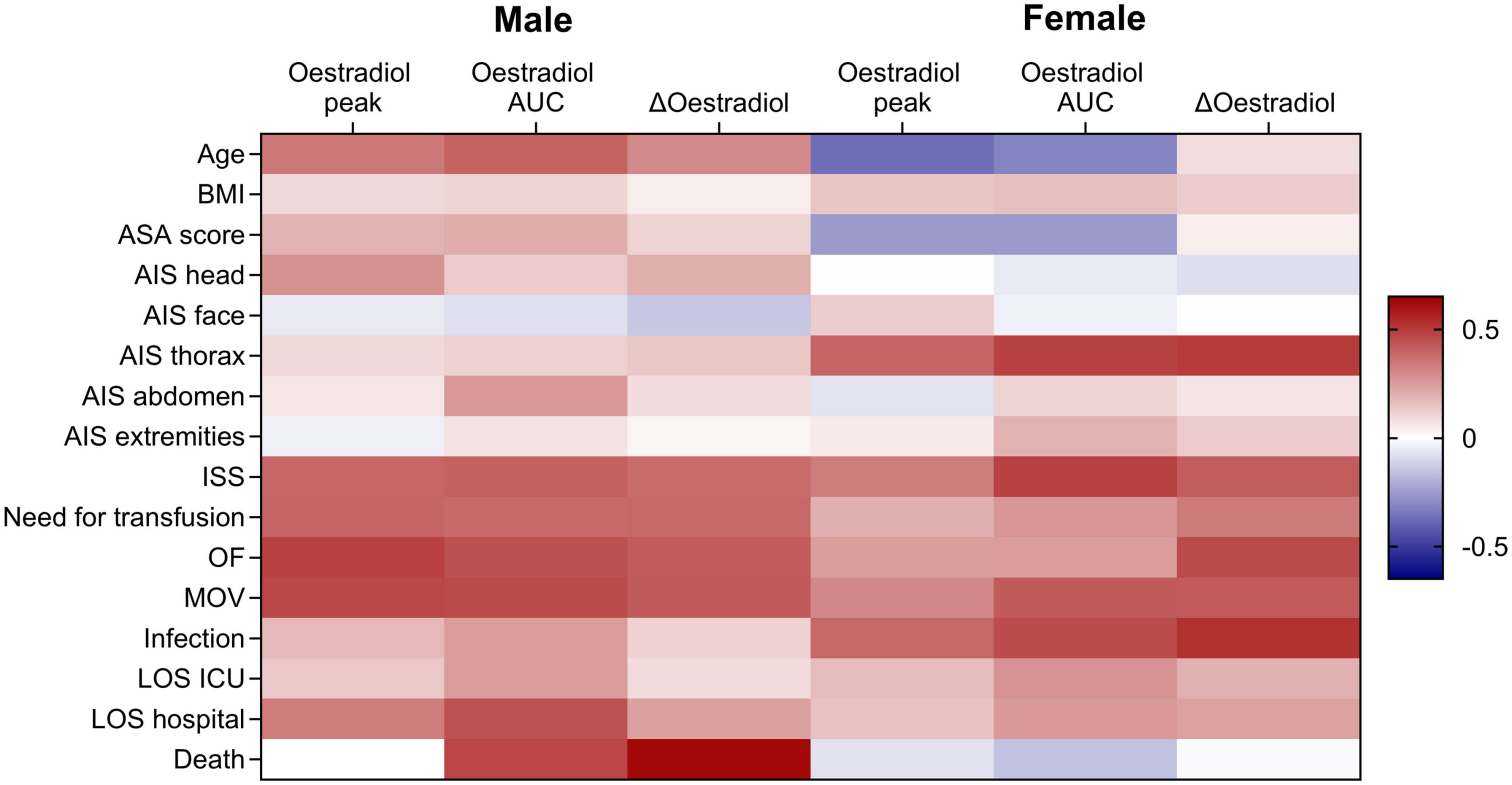
Sex-specific Spearman correlation heatmap of oestradiol parameters and clinical variables. The heatmap depicts correlation coefficients between oestradiol summary measures (peak concentration, area under the curve [AUC], and absolute change from baseline [Δ]) and selected demographic, injury-related, therapeutic, and outcome variables in male (left) and female (right) patients. Colours indicate the strength and direction of correlations using a fixed colour scale from blue to red (–0.65 to +0.65).

Correlation analyses were performed to explore associations between oestradiol summary measures and clinical parameters, stratified by biological sex (**Figure 2**). The full correlation matrix of all analysed variables is provided in the Supplementary Material (**Supplementary Figure 1**).

In men, oestradiol peak, AUC and Δ correlated positively with injury severity and adverse outcomes, with particularly strong associations for ISS, OF, mortality, and LOS hospital. In women, oestradiol parameters showed similar but less consistent patterns. AUC was most strongly associated with ISS, OF, MOF, and infectious complications, and positive correlations were also observed with AIS thorax. Associations with age, BMI, and ASA score were weak or inconsistent.

### 3.4 Association between oestradiol and injury severity

**Figure 3:**
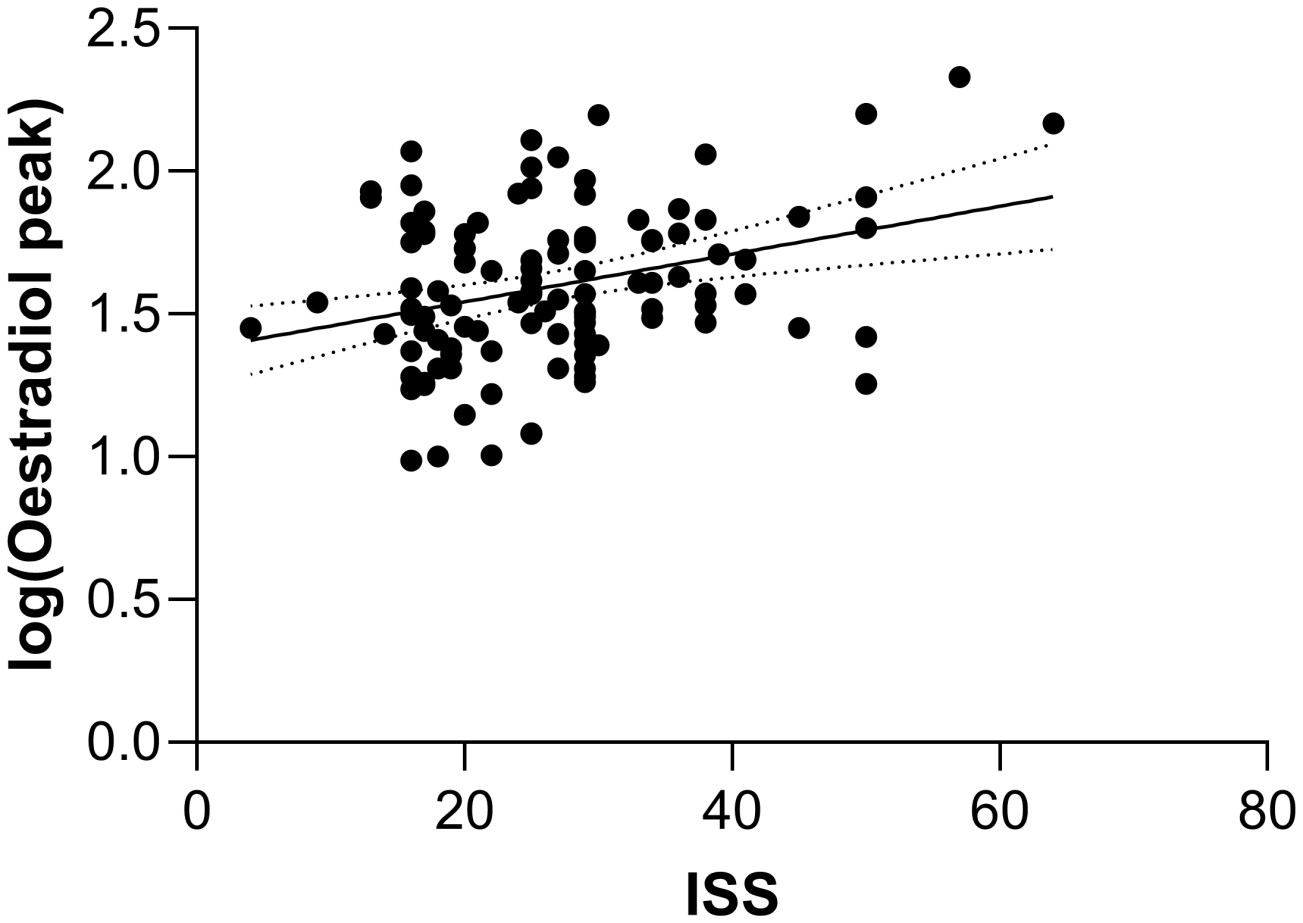
Correlation between the Injury Severity Score (ISS) and oestradiol peak levels after polytrauma. Oestradiol peak concentrations log transformed. Each dot represents an individual patient. The solid line represents the linear regression fit with 95% CI (dotted lines). Correlation was assessed using Pearson’s correlation coefficient.

The relationship between oestradiol and injury severity was examined using scatter plots and correlation analyses. Oestradiol peak levels correlated with the ISS (Pearson’s r = 0.32, p < 0.001; **Figure 3**). Comparable results were obtained when oestradiol exposure was quantified using AUC or Δ (**Supplementary Table 1**). The observed associations remained consistent when Spearman’s rank correlation analysis was applied (data not shown).

In a multivariable linear regression model adjusted for age and sex, injury severity as quantified by the ISS remained independently associated with log-transformed oestradiol peak levels (β = 0.0084, 95% CI 0.0036–0.0132, p < 0.001; **Table 2**). The model explained 12.4% of the variance in oestradiol peak concentrations. Results were consistent after additional adjustment for body mass index and ASA score; the full model is provided in the Supplementary Material (**Supplementary Table 2**). Comparable results were obtained when oestradiol exposure was quantified using AUC (data not shown).

**Table 2:** Multivariable linear regression analysis of factors associated with oestradiol peak concentration.

Covariate	*β* (e*st*imate)	95% CI	*P*-value
ISS	0.0084	0.0036 to 0.0132	< 0.001
Age	0.0019	−0.0008 to 0.0045	0.164
Sex (male)	0.0765	−0.0391 to 0.1922	0.192

Regression coefficients (β estimates), 95% CI, and corresponding p-values are shown for the associations between oestradiol peak concentration and Injury Severity Score (ISS), age, and sex. Oestradiol peak concentrations were logarithmically transformed prior to analysis.

### 3.5 Association of oestradiol levels with adverse clinical outcomes

Associations between oestradiol metrics and adverse outcomes were analysed using logistic regression models for binary outcomes and linear regression models for log-transformed hospital length of stay. For each outcome, an ISS-only model (Model 1) and an ISS-adjusted model including oestradiol (Model 2) were fitted. Key associations, effect estimates, and model performance are summarised in **Table 3**, while full regression results including sample sizes and event numbers are provided in **Supplementary Table 3**. Due to incomplete longitudinal hormone measurements, sample sizes differed between analyses depending on the oestradiol metric. Corresponding sample sizes are reported for each analysis and in the Supplementary Material.

**Table 3:** Association between oestradiol metrics and adverse clinical outcomes after polytrauma, comparing Injury Severity Score (ISS)-only models (Model 1) with ISS-adjusted models including oestradiol (Model 2).

Oestradiol metric	Effect estimate (95% CI)	*P*-value	Model performance
Organ failure (OF)			
Peak oestradiol (log)	*OR* 28.2 (4.25-186.5)	< 0.001	ROC AUC 0.69 → 0.77
Oestradiol AUC (log)	*OR* 28.5 (4.32–188.2)	< 0.001	ROC AUC 0.70 → 0.79
Δoestradiol	*OR* 1.05 (1.02–1.08)	< 0.001	ROC AUC 0.69 → 0.80
In-hospital mortality			
Peak oestradiol (log)	*OR* 34.5 (3.40–351.3)	0.003	ROC AUC 0.61 → 0.74
Δoestradiol	*OR* 1.03 (1.01–1.05)	0.002	ROC AUC 0.61 → 0.76
Oestradiol AUC (log)	*OR* 7.71 (0.91–65.34)	0.061	ROC AUC 0.63 → 0.68
Hospital length of stay (LOS)			
Peak oestradiol (log)	*β* 0.894 (0.254–1.533)	0.007	Adj. *R²* 0.024 → 0.091
Oestradiol AUC (log)	*β* 1.261 (0.745–1.777)	< 0.001	Adj. *R²* 0.026 → 0.208

Effect estimates represent odds ratios (OR) from logistic regression models for organ failure (OF) and in-hospital mortality, and regression coefficients (β) from linear regression models for hospital length of stay (LOS). Peak oestradiol concentrations and oestradiol area under the curve (AUC) were log-transformed prior to analysis. Hospital LOS was analysed using log-transformed values (log[LOS + 1]). Model performance is reported as receiver operating characteristic area under the curve (ROC AUC) for logistic regression models, and as adjusted R² for linear regression models. Detailed regression outputs, including sample sizes, event numbers, and full model performance metrics, are provided in **Supplementary Table 3**.

#### 3.5.1 Organ failure

ISS alone was significantly associated with OF (OR 1.07–1.08 per point; p ≤ 0.001; n = 109–116). After adjustment for ISS, all oestradiol metrics remained independently associated with OF. Peak oestradiol and oestradiol AUC showed strong associations with increased odds of OF (OR 28.2 and 28.5; both p ≤ 0.001), while Δoestradiol was also significantly associated (OR 1.05 per unit; p ≤ 0.001). Inclusion of oestradiol metrics improved model fit and discrimination compared with ISS alone, with Δoestradiol showing the best overall model performance.

#### 3.5.2 In-hospital mortality

ISS alone was not significantly associated with in-hospital mortality (OR 1.03–1.04 per point; p = 0.11–0.17; n = 109–116; 17 events). After adjustment for ISS, higher peak oestradiol levels were strongly associated with increased odds of mortality (OR 34.5; 95% CI 3.40–351.3; p = 0.003). Δoestradiol was also independently associated with mortality (OR 1.03 per unit; 95% CI 1.01–1.05; p = 0.002), whereas oestradiol AUC showed only a borderline association (OR 7.71; 95% CI 0.91–65.34; p = 0.061). Inclusion of oestradiol metrics improved model performance, particularly for peak oestradiol and Δoestradiol.

#### 3.5.3 Hospital length of stay

ISS alone showed no significant association with hospital LOS. After adjustment for ISS, higher peak oestradiol levels were significantly associated with longer hospital stay (β = 0.894; 95% CI 0.254–1.533; p = 0.007; n = 93). Oestradiol AUC demonstrated an even stronger association (β = 1.261; 95% CI 0.745–1.777; p < 0.001; n = 100). In contrast, Δoestradiol was not significantly associated with hospital LOS (β = 0.004; 95% CI −0.002 to 0.011; p = 0.198; n = 93). Inclusion of peak oestradiol and AUC improved model fit, whereas Δoestradiol did not.

#### 3.5.4 Summary of outcome associations

Across outcomes, higher oestradiol concentrations and greater post-traumatic increases in oestradiol were consistently associated with adverse clinical outcomes. Associations were strongest for OF, with all oestradiol metrics remaining independently associated after adjustment for injury severity and significantly improving model performance. For mortality, peak oestradiol levels and Δoestradiol demonstrated significant associations despite limited event numbers, whereas oestradiol AUC showed only borderline effects. For hospital length of stay, peak oestradiol and oestradiol AUC, but not Δoestradiol, were independently associated with prolonged hospitalisation.

## 4 Discussion

### 4.1 Interpretation of the findings

The key finding of this study is that oestradiol dynamics are independently associated with injury severity and adverse clinical outcomes after polytrauma.

Following severe trauma, suppression of the hypothalamic–pituitary–gonadal axis with declining concentrations of progesterone, testosterone, LH, and FSH, alongside increased oestradiol levels, has been consistently reported (33–36). This study confirms these alterations and demonstrates a distinct temporal profile of oestradiol, characterised by an early rise, a peak on days 2–3, and a subsequent decline. The early increase in oestradiol despite suppressed LH and FSH is in line with previous reports suggesting a peripheral contribution, potentially involving cytokine-mediated upregulation of aromatase in extragonadal tissues (47). However, this interpretation does not exclude other regulatory pathways. In particular, ovarian steroidogenesis may also be influenced by gonadotropin-independent mechanisms, including local growth factors, neuropeptides, and neurotransmitters (50, 51). Moreover, activation of the hypothalamic–pituitary–adrenal axis after severe trauma may alter adrenal steroidogenesis and the availability of androgenic substrates for peripheral oestrogen formation (52). The subsequent decline in oestradiol concentrations may reflect resolution of the initial inflammatory response or reduced availability of androgenic substrate. The observed temporal pattern is consistent with these mechanisms and existing clinical data (33, 43). Notably, the consistent timing of the oestradiol peak (day 2 in men, day 3 in women) may define a relevant window for future mechanistic and clinical studies.

Against this background, the observed associations with injury severity provide further insight into the clinical relevance of oestradiol dynamics. Positive correlations between oestradiol metrics and the ISS indicate that oestradiol may serve as an independent marker of systemic stress and inflammatory burden after severe trauma.

Beyond injury severity, oestradiol dynamics were associated with clinically relevant outcomes, with the strongest effects observed for OF. All oestradiol measures remained significantly associated with OF, and inclusion of oestradiol substantially improved model discrimination. These findings suggest that oestradiol reflects a clinically relevant dimension of post-traumatic systemic dysregulation and may contribute to the pathophysiology of organ dysfunction. Peak oestradiol and Δoestradiol were also significantly associated with in-hospital mortality, despite a limited number of events. Although confidence intervals were wide, the observed effect sizes indicate potential prognostic relevance. In contrast, oestradiol AUC showed only a borderline association with mortality, suggesting that early dynamics and peak values may be more informative than cumulative exposure. Peak oestradiol and AUC were further associated with prolonged hospital stay, whereas Δoestradiol was not, indicating that sustained elevation may be more relevant than the initial increase.

With regard to sex differences, our findings challenge the concept of a female-protective role of oestradiol after severe trauma, as previously suggested (34, 48). This is supported by largely similar oestradiol dynamics observed in men and women. The absence of sustained differences in oestradiol concentrations, together with comparable temporal profiles across sexes, suggests shared pathophysiological mechanisms. Although premenopausal women exhibited higher oestradiol concentrations at the initial assessment, these differences were no longer evident during the subsequent post-traumatic course, suggesting that trauma-associated changes in oestradiol concentrations may outweigh baseline differences related to menopausal status, during the early post-traumatic phase. As expected, testosterone, LH, and FSH differed between male and female patients, reflecting well-established physiological sex differences in reproductive hormone concentrations. However, the temporal trajectories of these hormones were broadly comparable between sexes, consistent with a trauma-induced suppression of the hypothalamic–pituitary–gonadal axis (33–36).

Clinical evidence on the role of oestradiol after polytrauma remains limited and methodologically heterogeneous. Previous studies have largely relied on single or very early hormone measurements and focused on isolated outcomes, predominantly mortality, without systematically accounting for individual hormone trajectories or objective measures of injury severity (33, 34, 42). In contrast, the present study is the first to provide a detailed longitudinal characterisation of reproductive hormone trajectories in a polytrauma cohort and to systematically examine multiple oestradiol parameters in relation to injury severity as quantified by the ISS and a range of clinically relevant outcomes.

Together with the direct association of oestradiol parameters and the ISS, these findings position oestradiol dynamics as a relevant component of the early systemic response to severe polytrauma and provide a robust basis for further mechanistic and prognostic investigations.

From a clinical perspective, oestradiol measurement represents an inexpensive and easily implementable biomarker approach, as assays are routinely available in most hospital laboratories and can be obtained from routinely collected blood samples. Given the observed associations with injury severity and adverse outcomes, serial assessment of oestradiol may provide complementary information for early risk stratification after polytrauma. However, external validation is required before clinical implementation.

### 4.2 Limitations

The design of this study entails several limitations that should be considered when interpreting the results. The investigation was conducted at a single Level 1 trauma centre, which may limit the generalisability of the findings to other settings or healthcare systems. Although the inclusion criteria and injury severity distribution are comparable to those of established trauma registries, external validation in independent, multicentre cohorts is warranted.

While the overall sample size was sufficient to detect clinically meaningful associations, the number of in-hospital deaths was limited. Although the observed event rate allowed for multivariable regression analyses with appropriate model complexity, mortality-related findings should be interpreted with caution and confirmed in larger cohorts. Furthermore, the number of premenopausal women was too small to allow meaningful or adequately powered comparisons between premenopausal and postmenopausal patients.

Hormone measurements were derived from routine clinical blood samples rather than study-specific sampling. While this approach reflects real-world clinical practice and enhances applicability, it may introduce pre-analytical variability related to sampling time, handling, or concurrent interventions that could not be fully controlled. In addition, hormone concentrations are subject to circadian variation, and samples were not consistently obtained at the same time of day, which may have introduced additional variability into the measurements. Furthermore, hormone measurements were not available at all predefined time points for all patients, resulting in varying sample sizes across analyses and oestradiol metrics. This may have introduced selection bias.

Despite adjustment for injury severity and selected covariates, residual confounding cannot be excluded, as is inherent to observational study designs. The menstrual cycle phase could not be fully accounted for in premenopausal women, and the small number of premenopausal participants limits conclusions regarding menopausal status.

### 4.3 Conclusions

In conclusion, this prospective study demonstrates that oestradiol exhibits a distinct post-traumatic temporal profile following severe polytrauma, differing fundamentally from the suppression observed in other reproductive hormones. Oestradiol concentrations were associated with the ISS and remained independently associated with OF, mortality, and hospital LOS after adjustment for ISS. These findings indicate that oestradiol dynamics convey clinically relevant information beyond established measures of trauma severity.

Together, the results challenge the concept of a uniformly protective role of oestradiol in the acute trauma setting and suggest that oestradiol may serve as both a biomarker of systemic stress and a potential modulator of post-traumatic pathophysiology. Further studies are required to validate these findings, elucidate underlying mechanisms, and determine the clinical utility of integrating oestradiol into prognostic models after polytrauma.

## Data Availability

The raw data supporting the conclusions of this article will be made available by the authors, without undue reservation.
