# Supplementary material for: Oestradiol dynamics after polytrauma are linked to injury severity and adverse outcomes
Source: Front Endocrinol (Lausanne). 2026 Aug 3;17:1878946. doi: 10.3389/fendo.2026.1878946 (PMC13478010; doi:10.3389/fendo.2026.1878946)
Supplement: Supplementary file 1 [file Supplementaryfile1.docx]

Supplementary Material

# 1 Questionnaire for the assessment of hormonal status after polytrauma

1. Have you had menstrual bleeding within the past 12 months? [yes/no]

If no:
a. I have had my uterus removed (hysterectomy). [yes/no]
b. I have undergone endometrial ablation. [yes/no]
c. Both ovaries have been removed. [yes/no]
d. I am using a progestin-releasing intrauterine device (IUD). [yes/no]
e. Other known reason: [free-text field]

2. For premenopausal women:

a. What was the first day of your last menstrual period? [date]
b. Is your menstrual cycle regular? [yes/no]
c. What is the usual length of your menstrual cycle? [days]

3. For postmenopausal women:

a. When was your last menstrual bleeding? [month/year]

4. For women:

a. Gravidity [number of pregnancies]
b. Parity [number of deliveries]

5. Which medications are you currently taking?

a. Complete list [free-text field]
b. Are you currently taking hormonal medications (e.g. oral contraceptives, menopausal estrogen therapy, etc.)? [yes/no]

i. If yes, which medication? [free-text field]
ii. Dosage [free-text field]
iii. Since when [month/year]

c. Have you taken hormonal medications in the past? [yes/no]

6. Do you currently have or have you previously had a malignant disease (cancer)? [yes/no]

a. If yes, type of tumour [free-text field]
b. Date of initial diagnosis [date]
c. Treatment [free-text field]

7. Do you have a history of pituitary gland (hypophysis) disease? [yes/no]

a. If yes, since when? [month/year]
b. What treatment was performed? [free-text field]

8. For men:

a. Are you currently taking steroid hormones or have you taken steroid hormones in the past? [yes/no]

i. If yes, which substances? [free-text field]
ii. Frequency? [free-text field]
iii. Dosage? [free-text field]
iv. Reason? [free-text field]

# 2 Temporal dynamics of Oestradiol concentrations after polytrauma

*Figure S1. Temporal dynamics of Oestradiol concentrations after polytrauma, stratified by menopausal status. Peripheral blood concentrations of 17β-oestradiol were measured on admission (day 0) and daily on days 1–5 in female patients. Premenopausal and postmenopausal women were analysed separately. Data are shown as mean ± SEM. Longitudinal changes were analysed using repeated-measures models. Asterisks indicate statistically significant differences between premenopausal and postmenopausal women at the respective time points (*p < 0.05); “ns” denotes non-significant differences.*

# 3 Full Spearman correlation heat maps

*Figure S2. Full Spearman correlation matrices for male and female trauma patients. The heatmaps display correlation coefficients between the oestradiol hormone parameters peak, area under the curve [AUC], and absolute change from baseline [Δ]) and clinical, injury-related, and outcome variables, shown separately for men and women. Colours indicate the strength and direction of correlations, ranging from negative (blue) to positive (red) on a scale from -0.65 to 0.65. The figure is provided for exploratory purposes and to complement the focused analyses presented in Figure 2.*

# 4 Correlation of oestradiol measures with injury severity

*Table S1. Correlation analysis between oestradiol summary measures and injury severity score (ISS).*

| Variable pair | Pearson’s *r* | *p*-value |
| --- | --- | --- |
| ISS – Oestradiol peak | 0.32 | < 0.001 |
| ISS – Oestradiol AUC | 0.37 | < 0.001 |
| ISS - ΔOestradiol | 0.39 | < 0.001 |

*Pearson correlation coefficients (*r*) and corresponding* p*-values are shown for the associations between ISS and oestradiol peak concentration, oestradiol area under the curve (AUC), and absolute change from baseline (Δ). Oestradiol concentrations were logarithmically transformed prior to analysis.*

# 5 Extended multivariable linear regression model for oestradiol peak levels

*Table S2. Multivariable linear regression analysis including additional covariates.*

| Covariate | *β* (estimate) | 95% CI | *p*-value |
| --- | --- | --- | --- |
| ISS | 0.0084 | 0.0036 to 0.0132 | < 0.001 |
| Age | 0.0019 | −0.0008 to 0.0045 | 0.164 |
| Sex (male) | 0.0765 | −0.0391 to 0.1922 | 0.192 |
| BMI | 0.0102 | -0.0019 to 0.0222 | 0.097 |
| ASA score | -0.0067 | -0.0898 to 0.0763 | 0.872 |

*Multivariable linear regression model examining the association between oestradiol peak concentration and Injury Severity Score (ISS) after additional adjustment for body mass index (BMI) and American Society of Anesthesiologists physical status classification (ASA score). Regression coefficients (β estimates), 95% CIs, and corresponding* p*-values are reported. Oestradiol peak concentrations were logarithmically transformed prior to analysis.*

# 6 Full regression analyses of oestradiol metrics and adverse clinical outcomes after polytrauma

*Table S3. Full regression analyses of the associations between oestradiol metrics and adverse clinical outcomes after polytrauma.*

**A) Organ failure (OF, logistic regression)**

|  | Model | Predictor | *OR* | 95 % CI | *p*-value | *n* | Events | AIC | ROC AUC |
| --- | --- | --- | --- | --- | --- | --- | --- | --- | --- |
| Peak oestradiol | | |  |  |  |  |  |  |  |
|  | Model 1 | ISS | 1.07 | 1.03 – 1.12 | 0.001 | 109 | 46 | 140.1 | 0.69 |
|  | Model 2 | ISS | 1.06 | 1.01 – 1.11 | 0.014 | 109 | 46 | 127.7 | 0.77 |
|  |  | Peak oestradiol (log) | 28.2 | 4.25 – 186.5 | <0.001 | 109 | 46 |  |  |
| Oestradiol AUC | | |  |  |  |  |  |  |  |
|  | Model 1 | ISS | 1.08 | 1.04 – 1.13 | <0.001 | 116 | 46 | 144.6 | 0.70 |
|  | Model 2 | ISS | 1.06 | 1.02 – 1.11 | 0.010 | 116 | 46 | 96.4 | 0.68 |
|  |  | Oestradiol AUC (log) | 28.5 | 4.32 – 188.2 | <0.001 | 116 | 46 |  |  |
| ΔOestradiol | | |  |  |  |  |  |  |  |
|  | Model 1 | ISS | 1.07 | 1.03 – 1.12 | 0.001 | 109 | 46 | 140.9 | 0.69 |
|  | Model 2 | ISS | 1.07 | 1.01 – 1.12 | 0.014 | 109 | 46 | 121.9 | 0.80 |
|  |  | ΔOestradiol | 1.05 | 1.02 – 1.08 | < 0.001 | 109 | 46 |  |  |

**B) In-hospital mortality (logistic regression)**

|  | Model | Predictor | *OR* | 95 % CI | *p*-value | *n* | Events | AIC | ROC AUC |
| --- | --- | --- | --- | --- | --- | --- | --- | --- | --- |
| Peak oestradiol | | |  |  |  |  |  |  |  |
|  | Model 1 | ISS | 1.03 | 0.99 – 1.08 | 0.166 | 109 | 17 | 96.5 | 0.61 |
|  | Model 2 | ISS | 1.00 | 0.95 – 1.06 | 0.890 | 109 | 17 | 88.1 | 0.74 |
|  |  | Peak oestradiol (log) | 34.5 | 3.40 – 351.3 | 0.003 | 109 | 17 |  |  |
| Oestradiol AUC | | |  |  |  |  |  |  |  |
|  | Model 1 | ISS | 1.04 | 0.99 – 1.09 | 0.111 | 116 | 17 | 98.2 | 0.63 |
|  | Model 2 | ISS | 1.02 | 0.97 – 1.07 | 0.512 | 116 | 17 | 96.4 | 0.68 |
|  |  | Oestradiol AUC (log) | 7.71 | 0.91 – 65.34 | 0.061 | 116 | 17 |  |  |
| ΔOestradiol | | |  |  |  |  |  |  |  |
|  | Model 1 | ISS | 1.03 | 0.99 – 1.08 | 0.166 | 109 | 17 | 96.5 | 0.61 |
|  | Model 2 | ISS | 0.99 | 0.94 – 1.05 | 0.755 | 109 | 17 | 87.5 | 0.76 |
|  |  | ΔOestradiol | 1.03 | 1.01 – 1.05 | 0.002 | 109 | 17 |  |  |

**C) Hospital length of stay (linear regression on log[hospital LOS + 1])**

|  | Model | Predictor | *β* | 95 % CI | *p*-value | *n* | Adj. *R²* | AIC |
| --- | --- | --- | --- | --- | --- | --- | --- | --- |
| Peak oestradiol | | |  |  |  |  |  |  |
|  | Model 1 | ISS | 0.014 | −0.002 – 0.030 | 0.076 | 93 | 0.024 | 224.5 |
|  | Model 2 | ISS | 0.009 | −0.007 – 0.024 | 0.274 | 93 | 0.091 | 218.9 |
|  |  | Peak oestradiol (log) | 0.894 | 0.254 – 1.533 | 0.007 | 93 |  |  |
| Oestradiol AUC | | |  |  |  |  |  |  |
|  | Model 1 | ISS | 0.015 | −0.001 – 0.030 | 0.061 | 100 | 0.026 | 238.2 |
|  | Model 2 | ISS | 0.003 | −0.012 – 0.017 | 0.705 | 100 | 0.208 | 218.5 |
|  |  | Oestradiol AUC (log) | 1.261 | 0.745 – 1.777 | <0.001 | 100 |  |  |
| ΔOestradiol | | |  |  |  |  |  |  |
|  | Model 1 | ISS | 0.014 | −0.002 – 0.030 | 0.076 | 93 | 0.024 | 224.5 |
|  | Model 2 | ISS | 0.011 | −0.006 – 0.027 | 0.192 | 93 | 0.031 | 224.8 |
|  |  | ΔOestradiol | 0.004 | −0.002 – 0.011 | 0.198 | 93 |  |  |

*The table reports detailed results from regression analyses for all outcomes. For each outcome, two models were fitted: Model 1, including Injury Severity Score (ISS) only, and Model 2, including ISS and one oestradiol metric. Each oestradiol metric (peak, area under the curve [AUC], and absolute change [Δ]) was analysed in a separate Model 2 to avoid overfitting. Binary outcomes (organ failure [OF] and in-hospital mortality) were analysed using logistic regression, and hospital length of stay (LOS) was analysed using linear regression on log-transformed values (log[LOS + 1]). Outcome parameters Akaike information criterion (AIC) and receiver operating characteristic area under the curve (ROC AUC) are given. The table is presented in three parts: A) OF; B) Mortality; C) Hospital length of stay.*
